# Fistules scrotales révélant un adénocarcinome mucineux du scrotum: à propos d’un cas

**DOI:** 10.11604/pamj.2017.26.190.9515

**Published:** 2017-03-30

**Authors:** Abdelilah El Alaoui, Hicham El Boté, Oussama Ziouani, Oussman Dembele, Hachem El Sayegh, Ali Iken, Lounis Benslimane, Yassine Nouini

**Affiliations:** 1 Service d’Urologie A, Hôpital Ibn Sina, CHU Rabat, Maroc

**Keywords:** Fistule, adénocarcinome mucineux, scrotum, canal anal, Fistula, mucinous adenocarcinoma, scrotum, anal canal

## Abstract

Les fistules scrotales sont rares, et souvent secondaires à des lésions de tuberculose. Les adénocarcinomes mucineux sont des tumeurs qui renferment au moins 50% de mucus extracellulaire, et siègent préférentiellement sur le recto sigmoïde, la localisation scrotale est inhabituelle. Nous rapportons un cas d’adénocarcinome mucineux secondaire du scrotum révélé par des fistules scrotales, chez un patient âgé de 54 ans, sans antécédents pathologiques notables, qui présente depuis deux ans des fistules scrotales à répétition, sans autres signes associés. L’examen trouve de multiples fistules scrotales avec issue de pus épais, le toucher rectal est normal. Les explorations urologiques (UIV, UCRM, Cystoscopie, …) sont normales, le bilan infectieux ainsi que la recherche de BK dans les urines et le crachat sont négatives. La biopsie de la peau scrotale est revenue en faveur d’un adénocarcinome mucineux moyennement différencié, dont l’étude immun histochimique est en faveur d’une origine primitive colorectale. L’évolution a été marqué par l’apparition de fistules anales complexes, ayant a l’IRM une caractéristique active, alimentant plusieurs collections pelvi périnéales comportant des bourgeons charnus. Sur le plan thérapeutique et vu la rareté de ce cancer, il n’y a pas de consensus. La résection chirurgicale reste le traitement de choix pour cette affection. La radio chimiothérapie pré opératoire est préconisée pour ce type de cancer mais son rôle n’est pas bien établi. Chez notre patient on a opté pour une radio chimiothérapie néo adjuvante première, avant la réalisation d’une amputation abdomino- périnéale.

## Abstract (en)

Scrotales fistulas are rare and often represent secondary lesions of tuberculosis. Mucinous adenocarcinomas are tumors containing at least 50% of extracellular mucus. They occur most commonly in the rectosigmoid; scrotal location is rare. We report the case of a 54-year old patient, with no particular past medical history, with secondary mucinous adenocarcinoma of the scrotum revealed by scrotal fistulas. The patient had suffered from recurrent scrotal fistulas for two years, with no other associated signs. Clinical examination showed multiple scrotal fistulas with discharge of thick pus. Rectal examination was normal. Urological examinations (IVU, UCRM, cystoscopy, …) were normal, the assessment of infectious diseases as well as the detection of BK virus in urine and sputum were negative. Biopsy of sample of scrotal tissue was in favor of moderately differentiated mucinous adenocarcinoma. Immunohistochemical examination was in favor of primary colorectal cancer. Patient’s evolution was marked by the occurrence of complex anal fistulas, which appeared on MRI as active, supplying several pelvic peritoneal collections resulting in fleshy buds. There is no consensus on the therapeutic approach due to the rarity of this cancer. Surgical resection is the treatment of choice for this disease. Preoperative chemotherapy and radiation therapy are recommended for this type of cancer, but their role is not well established. The patient underwent primary neoadjuvant chemotherapy and radiation therapy, before abdominoperineal excision.

## Introduction

Les fistules scrotales sont rares, et souvent secondaires à des lésions de tuberculose. Les adénocarcinomes mucineux sont des tumeurs qui renferment au moins 50% de mucus extracellulaire, et siègent préférentiellement sur le recto sigmoïde [1], la localisation scrotale est inhabituelle, et aucun cas n’a été décrit dans la littérature. Nous rapportons un cas d’adénocarcinome mucineux secondaire du scrotum révélé par des fistules scrotales.

## Patient et observation

**Figure 1:**
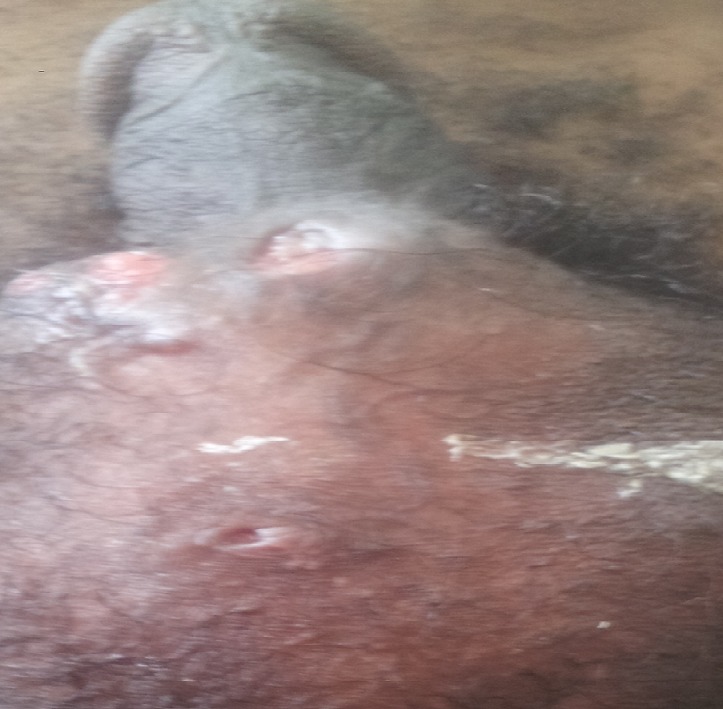
Fistules scrotales avec issue de pus et de mucus

**Figure 2:**
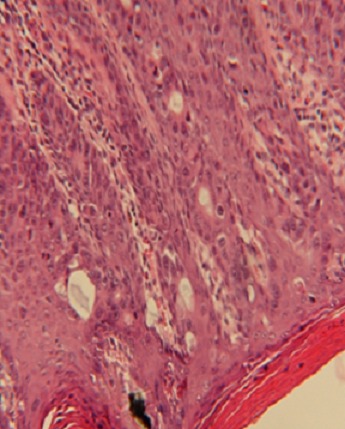
Aspect histologique de l’adénocarcinome mucineux

**Figure 3:**
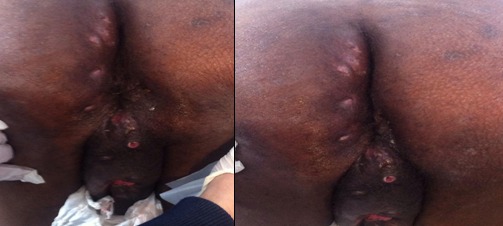
Plusieurs orifices fistuleux de part et d’autre de la marge anale, associés à des fistules scrotales avec issu de mucus

**Figure 4:**
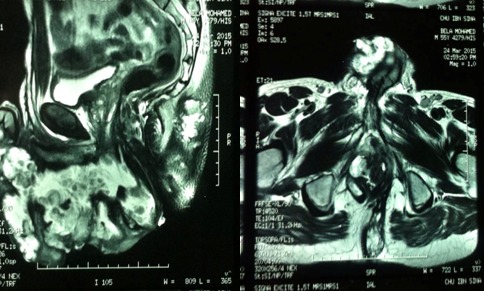
Fistules anales complexes alimentant plusieurs collections pelvi péritonéales comportant des bourgeons charnus avec épaississement ano rectal d’allure tumorale

Il s’agit d’un patient âgé de 54 ans, sans antécédents notables, notamment pas d’habitudes toxiques ni de notion de contage tuberculeux; présente depuis deux ans un écoulement purulent par de multiples orifices scrotaux, sans notion de trouble mictionnelle, le tout évoluant dans un contexte d’amaigrissement chiffré à 6 kg en 2 mois. L’examen des organes génitaux externes trouve de multiples fistules scrotales avec issue de pus épais (Figure 1), le toucher rectal est normal. le reste de l’examen clinique est normal, il n’y a pas d’adénopathies notamment au niveau de la région inguino-scrotale. Les explorations urologiques (UIV, UCRM, Cystoscopie,…) sont normales, le bilan infectieux ainsi que la recherche de bacilles de koch dans les urines et le crachat sont négatifs. La biopsie de la peau scrotale est revenue en faveur d’un adénocarcinome mucineux moyennement différencié (Figure 2), dont l’étude immun histochimique est en faveur d’une origine primitive colorectale. La coloscopie totale avec cathétérisme de la dernière anse iléale ne montre pas de lésions, l’évolution a été marquée par l’apparition de fistules anales complexes (Figure 3). L’IRM pelvienne révèle la présence d’un processus tissulaire ano rectal modérément hyper T2 dont le pôle inférieur est situé à proximité de la marge anale s’étendant environ sur 5 cm de la marge anale avec multiples trajets fistuleux (au moins 6) inter et trans sphinctériens en hyper signal T2 rehaussés après injection de produit de contraste (Figure 4). Le patient est adressé pour une radio chimiothérapie néo adjuvante première, avant la réalisation d’une amputation abdomino- périnéale.

## Discussion

Le carcinome du scrotum est une tumeur rare avec une incidence annuelle globale d´environ 1,5 par 1.000.000 personnes dans les pays occidentaux [2]. Les types histologiques les plus fréquents sont le carcinome épidermoïde, la maladie de Paget extra-mammaire, le carcinome baso-cellulaire, et le sarcome [3]. L’adénocarcinome primitif du scrotum est une tumeur très rare comme le témoin le peu d’observations rapportés dans la littérature [4, 5], alors que pour celui du carcinome mucineux aucun cas n’a été décrit, les localisations métastatiques au niveau du scrotum même rare, des cas de carcinome urothéliale, d’adénocarcinome du tractus digestif et bilio pancréatique ont été rapportés [4]. Chez notre patient, un adénocarcinome mucineux du scrotum a été retrouvé lors de bilan des fistules scrotales récidivantes, dont l’étude immun histochimique était en faveur d’une origine primitive colorectale. L’apparition par la suite de fistules anales, et le résultat de l’IRM pelvienne qui a objectivé de multiples fistules anales complexes, avec un processus tissulaire ano-rectal ont évoqué une origine primitive anale, malgré la normalité de la colonoscopie. Les adénocarcinomes mucineux du canal anal associés à des fistules périnéales chroniques sont des tumeurs très rares qui posent des problèmes diagnostiques et thérapeutiques [6], leur symptomatologie clinique n’est pas spécifique, elle est faite de rectorragies et de proctalgies, ce qui explique le retard diagnostique; cependant il y‘a un signe important c’est l’issu du mucus par les orifices fistuleux. Le diagnostic de certitude nécessite un examen histologique du trajet fistuleux avec étude immuno histochimique, cette dernière a permis de confirmer le diagnostic chez notre patient. L’IRM pelvienne est l’examen de choix pour l’exploration de cette tumeur en étayant une cartographie des lésions par rapport aux sphincters et aux muscles [7]. Sur le plan thérapeutique et vu la rareté de ce cancer, il n’y a pas de consensus. La résection chirurgicale (AAP) reste le traitement de choix pour cette affection. La radio chimiothérapie pré opératoire est préconisée pour ce type de cancer mais son rôle n’est pas bien établi [8], des études complémentaires sont nécessaires afin de standardiser le traitement. Le pronostic de cette affection dépend du retard diagnostique; il est variable selon les auteurs, le taux de survie à 5 ans est de 4,8% selon Jensen [9], et de 12 à 42% selon Moller [10]. Quand au risque de récidive il est faible après un délai de trois ans sans récidive.

## Conclusion

Le carcinome mucineux sur fistule scrotale et périnéale est une entité rare d’évolution grave d’où la nécessité de faire le diagnostic à un stade précoce. Toute fistule chronique de présentation inhabituelle doit faire évoquer la possibilité de ce cancer et de pratiquer une analyse histologique du trajet fistuleux.
